# The Effect of Women’s Differential Access to Messages on Their Adoption of Mobile Health Services and Pregnancy Behavior in Bangladesh: Retrospective Cross-Sectional Study

**DOI:** 10.2196/17665

**Published:** 2020-07-20

**Authors:** Mafruha Alam, Cathy Banwell, Kamalini Lokuge

**Affiliations:** 1 Australian National University ACT Australia

**Keywords:** mHealth, inequality, access, pregnancy

## Abstract

**Background:**

Text or voice messages have been used as a popular method for improving women’s knowledge on birth preparedness and newborn health care practices worldwide. The Aponjon service in Bangladesh provides twice-weekly messages to female subscribers about their pregnancy and newborn care on mobile phones that they own or share with family members. It is important to understand whether women’s singular access to a phone affects their service satisfaction and the adoption of health messages before deploying such interventions in resource-limited settings.

**Objective:**

This study aims to evaluate the effect of women’s singular and shared access to mobile phone messages on their service utilization and perceived behavioral change around birth preparedness and pregnancy care.

**Methods:**

In 2014, Aponjon conducted a retrospective cross-sectional survey of 459 female subscribers who received text or voice messages during their pregnancy by themselves (n=253) or with family members (n=206). We performed multivariable regression analyses to investigate the association between pregnant women’s differential access to messages and other socioeconomic factors and outcomes of service satisfaction, ability to recall service short code, ability to identify danger signs of pregnancy, preference for skilled delivery, arrangement of a blood donor for delivery and pregnancy complications, maternal nutrition, use of potable drinking water, and washing hands with soap for hygiene.

**Results:**

In the multivariable analysis, women who had singular access to messages had higher odds of reporting high satisfaction (odds ratio [OR] 1.72, 95% CI 1.12-2.63; *P*=.01), recalling the service short code (OR 2.88, 95% CI 1.90-4.36; *P*<.001), consuming nutritious food 5 times a day (OR 1.58, 95% CI 1.04-2.40; *P*=.03), and following the instructions of Aponjon on drinking potable water (OR 1.90, 95% CI 1.17-3.09; *P*=.01) than women who shared access with family members. Women’s differential access to messages did not affect their knowledge of danger signs and preparedness around delivery. Adolescent women and women aged 20-24 years had lower odds of planning safe deliveries than older women (aged≥25 years). Secondary education was statistically significantly associated with women’s ability to recall the short code and pregnancy danger signs, plan safe delivery, and select blood donors for emergencies. Higher family income was associated with women’s satisfaction, recognition of danger signs, and arrangement of blood donors and nutritious diet. Women who received more than 4 antenatal care visits had higher odds of liking the service, preferring skilled delivery, recalling danger signs, and consuming nutritious food.

**Conclusions:**

The capacity of women to independently access mobile phone messages can improve their adoption of mobile health services and some pregnancy health care practices. A holistic approach and equitable support are required to improve access to resources and knowledge of delivery preparedness among low-literate and younger women in low-income households.

## Introduction

### Background

The World Health Organization (WHO) defined mobile health (mHealth) as a medical and public health practice that is supported by mobile devices and other wireless devices [1]. Many developing countries around the world have recognized that mHealth can provide crucial lifesaving information in remote settings where the health workforce is scarce and have adopted appropriate mHealth interventions [1]. Mobile phone reminders and text messages have helped improve facility utilization for maternal and neonatal care in developing countries [2,3]. However, mHealth implementers have had difficulty reaching out to women who had no access to phones [2]. Approximately 21% of women worldwide have no phone, mainly because of cost, husband’s disapproval, and lack of technical knowledge on how to operate a phone [4]. Women’s non–phone ownership is most prevalent in Africa, the Middle East, and South Asia [5], regions known to contribute heavily to the global burden of maternal and neonatal deaths [6-8].

Bangladesh, a South Asian country with a population of approximately 160 million people in an area of 147,570 km^2^, has made significant progress in reducing the under-5 mortality rate and the maternal mortality ratio (MMR), which used to be one of the highest in the world [9,10]. However, MMR and neonatal mortality rate (NMR) remain at 170 per 100,000 live births and 23 per 1000 live births, respectively, which are far behind the targets set by the millennium development goals [9,10]. Similar to other member countries of the United Nations, Bangladesh needs continued investment and innovative approaches to reduce the MMR to 70 per 100,000 live births and NMR to 12 per 1000 live births, including improving health and well-being of all people of all ages by 2030 under the postmillennium sustainable development agenda [11,12]. mHealth initiatives could boost Bangladesh’s progress as mobile phone subscription has increased exponentially since its introduction in the early 90s [13]. Low-income households in rural settings are increasingly subscribing to mobile phones because of competitive pricing among telecom operators and mobile phone manufacturers [13]. Although the gender gap in mobile phone ownership is closing, it still exists. A study in rural Bangladesh revealed that ownership of mobile phones was almost 1.8 times more among men than women, and men were more likely to own a phone at an earlier age [14]. Women in low-income households are expected to share phones with family members more often than men [4,14,15]. Mobile phone acquisition was higher in households where only the husband was literate compared with households where only the wife was literate [13].

Inequity in access to technology such as mobile phones because of social, cultural, and economic differences is broadly known as the *digital divide* [13]. mHealth implementers in resource-limited settings need to address the *digital divide*, as women with shared or no access to phones and limited education may never receive health messages, especially voice messages that cannot be stored and read later like text messages [14-16]. In resource-limited settings where women do not have personal mobile phones, providing health workers or midwives with mobile phones for improved counseling and referrals has been tested [17,18]. However, this approach does not address the severe scarcity of skilled health workforce in remote areas [19]. As *phone sharing* is a common scenario in low-income households, it is important to understand how *shared access* to mHealth messages impacts women’s adoption of recommended maternal and newborn health and well-being practices [15]. Previous studies reported low to moderate changes in maternal facility utilization among women who had *any form of access* to mobile phones compared with women with *no access* [20-22]. There is a dearth of literature evaluating women’s *independent* and *shared* access to targeted messages on well-being practices during maternity.

### Objectives

To cover the gap in existing knowledge on the impact of the *digital divide* on pregnancy and birth preparedness, we conducted a study of female subscribers enrolled in a mobile phone–based health education messaging service during their pregnancy. The objective of our study was to investigate the association between how women accessed messages and their (1) satisfaction and intention to access the mHealth service and (2) their knowledge and practice around birth preparedness and pregnancy wellness.

## Methods

### The Intervention

The *Aponjon* (meaning someone very dear or close in Bangla) service was the first national mobile phone–based health education service for pregnant women and mothers of 0- to 11-month-old babies in Bangladesh that was deployed as the first project of the global initiative the Mobile Alliance for Maternal Action in 2012 and had received endorsement from the Bangladeshi government and financial support from the United States Agency for International Development [23,24]. The Aponjon service typically provides a range of maternal and newborn well-being information to registered Bangladeshi women according to their gestational stage [23]. Any pregnant woman can enroll in the service at any stage of her pregnancy to receive pregnancy messages and then in the postpartum service for another year after childbirth [23]. Usually during pregnancy or after childbirth, potential subscribers are contacted by community health workers of partner nongovernment organizations and included in the service with their consents [23,25]. Alternatively, potential subscribers can enroll in the service by directly calling the service short code, which has been advertised widely through television commercials, billboards, newspaper advertisements, and leaflets [23,25]. Ideally, it is expected that a pregnant woman will enroll in the service during pregnancy and, at the end of her pregnancy, will be upgraded to the maternal service for another year on successful birth of a child. Subscribers are reminded to contact the service call center by dialing the service short code for a service upgrade and complain or queries.

Pregnant women receive a range of information on birth preparedness, including recognition of danger symptoms of pregnancy, labor and delivery, decision making around delivery places and skilled birth attendants, arrangement of blood donors for complications during delivery and pregnancy-related emergencies, nutrition during pregnancy, hand washing procedure before eating, food preparation, and toilet and safe drinking water. The service was piloted for a year before rolling out in 2012 [23]. Subscriber women may choose between interactive voice resonance (IVR) and text messages as a service mode [25]. Each IVR message in Bangla is 1 min long, whereas text messages are in transliterated Bangla to support all mobile phone and contain up to 161 characters [25].

Aponjon female subscribers are enrolled in the service on confirmation that they have at least one mobile phone (own or shared) in their house that they can access at a certain time of the day. During enrollment, subscribers are asked to indicate the preferred time and days of the week to receive messages. The idea is that subscribers would have the handset for accessing messages at the specified time [23]. A female subscriber typically receives 2 messages a week from the service and an additional message for her husband or family member on a separate or the same mobile number if she includes them in the service [23,25]. The messages have been designed in accordance with the national and international guidelines on maternal, neonatal, and infant health care by accredited content experts [23,24].

Subscribers are typically charged 2.3 Bangladeshi Taka (BDT; US $0.03) per message with a *no fees* option for marginalized households [23,24]. Since mid-2013, the service has offered consultations with medically trained doctors through a 24-hour call center for clients [26]. Subscribers can consult doctors on maternal and newborn health issues for as long as they want, while being charged the normal call rate per minute [26]. In 2014, the service had more than one million subscriber bases [25].

### Study Area

We analyzed data obtained from a cross-sectional survey as part of routine operations research conducted by Dnet, the implementing agency of Aponjon. The survey was conducted between February and April 2014 in selected subdistricts of Bogura, Bagerhat, Patuakhali, Chittagong, and Laxmipur districts in Bangladesh, which were purposively selected to reflect the remoteness, cultural diversity, geographical dispersion, and maximum acquisition of subscribers [25]. Administratively, Bangladesh is divided into 8 divisions, 64 districts (*zillas*), and 545 subdistricts (*upazillas*) [9,27]. The subdistricts or *upazillas* are further divided into urban and rural areas; rural areas in an *upazilla* consist of union *parishads* (UP), whereas *mouzas* (cluster of villages) make up each UP [28,29]. Urban areas in an *upazilla* are divided into wards and *mahallas* (cluster of households) within wards [28,29]. Bogura is a northern district in Bangladesh, which is also known as the industrial powerhouse of the North Bengal [30]. Bagerhat and Patuakhali are 2 districts in southwestern Bangladesh and lie in the fringe of Bay of Bengal [31,32]. Chittagong (Chattagram) is a district in the southeastern part of Bangladesh, known for the sea port and hill tracts [33], whereas Laxmipur is a district in the southern part of Bangladesh [34]. The average size of households in these districts varied from 3.8 to 5.1 (Bogura: 3.8, Bagerhat: 4.13, Chittagong: 5.1, Laxmipur: 4.71, and Patuakhali: 4.41) in 2011 [30-34]. Among the selected districts, Bagerhat and Chittagong have the highest average literacy rate at 58.98% (male: 59.97% and female: 57.99%) and 58.90% (male: 61.1% and female: 56.7%), respectively [31,33].

### Sampling

The survey included subscriber women at different stages of pregnancy. Pregnant women were eligible to participate if they received pregnancy messages for at least two months and did not undergo a planned or unplanned pregnancy termination. Pregnant women who had just given birth to newborn baby but had not upgraded the service for the postpartum period were also eligible for the survey. Adolescent women aged less than 18 years were excluded from the survey.

A sampling frame with details of approximately 2274 potential survey respondents (who matched the inclusion criteria) was prepared from the Aponjon service database. The survey database contained information such as subscriber’s name, address, cell phone number, age, date of beginning of last menstruation period, enrollment date, and type of access and socioeconomic information. On the basis of formative research experience, Dnet estimated a priori that a sample size of approximately 400 respondents was required, with an anticipated ratio of shared to independent access of 1:2, to detect a difference in proportions with outcomes of interest between shared and independent access of 15%, with 80% power and a 5% significance level. Assuming availability of pregnant women at home and consent rate of 60%, it was expected that 660 subscribers would need to be contacted for the study.

Owing to the lack of availability of an adequate number of community health workers who could assist field researchers in identifying households of subscribers, only 839 subscribers were potentially available to be contacted from the list. Eligible subscribers were randomly sampled district by district from the existing list until the proposed number of respondents had been recruited. A group of 24 field researchers, 2 field supervisors, and 1 central coordinator conducted the survey. A pair of male and female researchers conducted each interview at the respondent woman’s house after ensuring privacy [25]. Each interview lasted approximately 1 hour. Before visiting the respondents at their home, researchers contacted the respondents over the phone to make an appointment. Initially, researchers read aloud information on the survey, confidentiality issues, benefits, and possible risks associated with participating in the survey. Respondents had the right to withdraw from the survey at any time and could refuse to answer any question. Verbal and written consents were received before each interview. Identification of respondents such as name, location, and cell phone number was replaced with IDs to maintain anonymity. The interviews were conducted in Bangla. Eligible respondents who could not be reached over the phone for an appointment or were not at home when field researchers visited or who did not want to participate were excluded from the survey [25]. Mostly, pregnant women who had relocated to have their birth at their parent’s house outside the study area could not be interviewed.

Respondents were administered semistructured questionnaires that contained questions regarding pregnancy behavioral outcomes as well as service-related questions such as how women accessed the service, satisfaction with the service, recall of service short code, and perceived knowledge and benefits from the service.

All research instruments and consent forms for this service were reviewed by an international institutional review board (IRB) and received an exemption from IRB review. The authors received approval to analyze the survey data of Aponjon service from Dnet, the implementing agency of Aponjon service, and the Science and Medical Delegated Ethics Review Committee of the Australian National University.

### Measures

The explanatory variable *type of access* was derived from the question, “Who accessed messages?,” with responses options being “me,” “me and family member,” or “family member.” We recorded the responses to *women=1* and *women or family member*=0. We hypothesized that women who accessed messages alone were more empowered and would access messages more regularly than women who shared access with family members, which would impact women’s adoption of recommended practices in these 2 groups.

Our outcome of interest, satisfaction of respondents, was measured by asking them to rate the service on a 5-point Likert scale, ranging from 1 (very bad) to 5 (excellent). We recoded *high satisfaction* by categorizing scores 4 and 5 as *high=1* and scores 1 to 3 as *low=0*.

The outcome variable *recalls short code* was determined from a service-related question, “Please tell me the service short code (the number you see flash on your mobile phone when Aponjon messages come).” Respondents who were able to recall the 5 digits correctly were coded as *knows short code=1* and who could not were codes as *does not know short code=0.* We considered this variable to demonstrate women’s familiarity with the service.

We measured the respondents’ knowledge of pregnancy danger signs. They were asked, “Can you tell me the danger signs?” These included high fever, severe headache, blurry vision, convulsions, vaginal bleeding, severe lower abdominal pain, swelling in hands and feet, hypertension, and less fetal movement. We generated a binary variable *able to recall danger signs* defined as *yes=1* if respondents could recall at least two signs and *no=0* for respondents who could not recall.

WHO’s birth preparedness and complication readiness (BPCR) plan recommends that pregnant women and their families are aware of planning the following elements before delivery: the place of birth, the birth attendant who will provide assistance during delivery, the location of the closest facility for delivery and emergency, arrangement of funds for any expenses related to delivery and emergency, supplies and materials necessary to bring to the facility, an identified labor and birth companion, an identified person who will look after the home and other children while pregnant woman is away, arrangement of transport to a facility for delivery and emergency, and the identification of compatible blood donors for delivery and emergency [35]. The outcome variable regarding birth preparedness around delivery for our study was derived from 3 sequential questions asked to pregnant women. The first question was, “Have you decided where your baby will be born?,” with 2 responses *yes* or *no*, and the second question for women who responded *yes* to first question, “(If decided) where do you want your baby to be born?” with 2 possible responses *hospital or health facility* and *home*. The third question was, “(For home based delivery) who will deliver your baby?,” with 2 responses *untrained birth attendant/relatives* and *trained birth attendant*. Responses to these 3 questions were grouped into binary responses as *Planned skilled delivery=1* and *Planned untrained birth attendants at home or made no plans yet=0*.

Outcome variable “selected blood donor for delivery or pregnancy emergency” was derived from the question, “Did you select a blood donor for delivery or complications during pregnancy?” Responses such as “yes I did” was coded as *yes=1*, and other responses such as “no,” “I did not know,” “haven’t thought about it,” or “I don’t find it’s necessary” were coded as *no=0*.

Having a balanced diet with essential macro and micronutrients is immensely important for pregnant women. Malnourished mothers are likely to give birth to premature babies with low birth weights who are at risk of dying within the first week of birth [9,36,37]. A maternal nutritional behavior–related binary outcome variable *consumed nutritious food 5 times a day* was derived from the question, “How many times in a day you eat one of these food items-vegetables, fruits, protein (such as milk, fish, meat, chicken, egg)?,” with count responses such as 1, 2, 3, 4, 5, and 6. Reponses 5 and above were recoded as *consumed nutritious food 5 times a day=1*, and responses less than 5 were recoded as *no=0*.

Bangladesh, similar to other South Asian countries, has a supply of poor-quality drinking water, which is contaminated with microbial pathogens and pollutants (such as arsenic) that are responsible for diarrheal and other infectious diseases [38]. Aponjon advises female subscribers to ensure a clean source of drinking water and treat the water before drinking. A binary variable “followed Aponjon’s instruction on drinking water” was constructed from 2 questions. The first question was, “What is your source of drinking water?,” with 2 responses “I drink water that has been properly treated with various methods (such as boiling, filter)” and “I drink surface water directly from source (such as tap, pond).” The second question was, “Where did you learn about treating drinking water?,” with 2 response options *Aponjon* and *Other*. The responses of these 2 questions were grouped into *yes=1* and *no=0* for our outcome variable.

Aponjon provides information on washing hands properly before food handling, taking meals, and after cleaning body parts. Women were asked, “How do you clean your hands for hygiene purposes?,” with possible answers such as “only water” and “with soap or disinfectants.” Women who replied “with soap or disinfectants” were asked, “Where did you learn to wash your hands in this way?,” with possible answers “Aponjon,” “Aponjon and others,” and “other sources.” Women who answered “Aponjon” or “Aponjon and others” were classified as “followed Aponjon’s hand-washing procedure=1” and who answered “other sources” as “followed hand-washing procedure learnt from other sources=0.”

Other covariates considered for this study were respondent women’s age (<20, 20-24, and ≥25 years), education (none or primary, junior secondary, and secondary or above), family income (BDT ≤10,000; 10,0001-20,000; and >20,000), first-time pregnancy (yes or no), place of residence (urban and rural), and districts (A, B, C, D, and E). We labeled district names to maintain anonymity. We also considered the frequency of antenatal care (ANC) visits (>4 ANC visits and ≤4 ANC visits) for all models. All these variables were selected because of their importance in the study or because of their demonstrated association with maternal and newborn health-related outcomes in mHealth interventions in previous research [9,20].

### Statistical Analysis

The respondent characteristics are described using frequencies and percentages overall and by differential access groups (*women* and *women or family members*). Chi-squared tests were performed to examine the distribution of sample background characteristics and outcome variables by explanatory variables (who accessed messages).

We undertook multiple multivariable logistic regression analysis to investigate the relationship between *who accessed messages* and the outcomes of interest. Multicollinearity tests were performed using the variance inflation factor (VIF) test to assess the correlation between covariates. A VIF score of greater than 2 was set as a threshold, and variables at this threshold were dropped from the final models. We performed logistic regression for the following outcomes of interest: *high satisfaction on the service*, *recalled short code*, *planned delivery by skilled attendant at home or a hospital*, *able to recall danger signs*, *selected a blood donor for delivery or pregnancy complications*, *consumed nutritious food 5 times a day*, *followed Aponjon instruction on potable drinking water*, and *followed Aponjon’s instruction on hand-washing*. Multivariable models were adjusted for women’s age, education, family income, first pregnancy, ANC visits, place of residence, and district (to account for the sampling strategy). The Hosmer-Lemeshow test was used to test the goodness of fit for the models, and a model was considered a good fit when the *P* value was nonsignificant. Statistical significance was determined at a *P* value of less than .05. We used IBM SPSS Statistics for Windows version 24.0 for the analysis. The results are expressed as adjusted odds ratio (OR) with 95% CIs.

## Results

### Background Information of Respondents

Of the 687 subscribers contacted, approximately 66.8% (459/687) of them who had a successful live birth recently (209/459, 45.5%) or were in different stages of pregnancy (250/459, 54.5%) were interviewed. Socioeconomic characteristics of respondents are described in Table 1. Women’s differential access to messages showed statistically significant differences in women’s educational levels and districts. Approximately 98.9% (454/459) of women received voice messages over text. The outcome variables are described in Table 2.

**Table 1:** Background characteristics of participants by access to messages (N=459).

Variables		Who accessed messages		*P* value
		Women (n=253), n (%)	Women or family member (n=206), n (%)	
**Age of respondents (years)**				.35
	<20	41 (16.2)	44 (21.4)	
	20-24	127 (50.2)	95 (46.1)	
	≥25	85 (33.6)	67 (32.5)	
**Educational qualification of women**				.005
	None or primary education	77 (30.4)	88 (42.7)	
	Junior secondary education	90 (35.6)	73 (35.4)	
	Secondary school or higher	86 (34.0)	45 (21.9)	
**Family monthly income, BDT^a^** **(US $)**				.10
	≤10,000 (118.15)	105 (41.5)	100 (48.5)	
	10,000-20,000 (118.15-236.30)	95 (37.5)	87 (42.2)	
	>20,000 (236.30)	53 (21.0)	19 (9.3)	
**First-time pregnancy**				.97
	Yes	131 (51.8)	107 (51.9)	
	No	122 (48.2)	99 (48.1)	
**Place of residence**				.99
	Urban	140 (55.3)	114 (55.3)	
	Rural	113 (44.7)	92 (44.7)	
**Districts**				.03
	District A	30 (11.9)	31 (15.0)	
	District B	68 (26.9)	39 (18.9)	
	District C	65 (25.7)	41 (20.0)	
	District D	36 (14.2)	29 (14.1)	
	District E	54 (21.3)	66 (32.0)	
**Number of antenatal care visits**				.42
	>4	153 (60.5)	132 (64.1)	
	≤4	100 (39.5)	74 (35.9)	

^a^BDT: Bangladeshi Taka.

**Table 2:** Unadjusted outcome variables by type of access.

Outcome variables		Who accessed messages		*P* value
		Women, n (%)	Women or family member, n (%)	
**High satisfaction (N=459)**				.004
	Yes	181 (71.5)	121 (58.7)	
	No	72 (28.5)	85 (41.3)	
**Recalled short code (n=458)**				<.001
	Yes	175 (69.4)	88 (42.7)	
	No	77 (30.6)	118 (57.3)	
**Able to recall danger signs (N=459)**				.19
	Yes	149 (58.9)	109 (52.9)	
	No	104 (41.1)	97 (47.1)	
**Planned skilled delivery (n=248)**				.77
	Yes	73 (49.7)	52 (51.5)	
	No	74 (50.3)	49 (48.5)	
**Identified blood donor (n=249)**				.03
	Yes	34 (22.8)	12 (12.0)	
	No	115 (77.2)	88 (88.0)	
**Consumed nutritious food 5 times a day (n=454)**				.04
	Yes	135 (54.4)	92 (44.7)	
	No	113 (45.6)	114 (55.3)	
**Followed instruction on potable drinking water (n=457)**				.005
	Yes	79 (31.2)	40 (19.6)	
	No	174 (68.8)	164 (80.4)	
**Followed Aponjon messages on hand washing** **(n=453)**				.31
	Yes	65 (26.1)	45 (22.1)	
	No	184 (73.9)	159 (77.9)	

### Effect on the Adoption of Mobile Health Services

Whether female subscribers were the sole receivers of the messages had statistically significantly increased their satisfaction with the service (OR 1.72, 95% CI 1.12-2.63; *P*=.01) and ability to recall the service short code correctly (OR 2.88; 95% CI 1.90-4.36; *P*<.001; Table 3).

Multivariable analysis of service satisfaction suggests that besides the *type of access*, women’s middle-income (OR 1.96, 95% CI 1.24-3.11; *P*=.004) and higher-income (OR 2.48, 95% CI 1.25-4.93; *P*=.009) background and completion of more than 4 ANC visits (OR 2.86, 95% CI 1.82-4.48; *P*<.001) significantly impacted their satisfaction with the service than their counterparts (Table 3). Similarly, covariates such as completion of secondary education (OR 2.28, 95% CI 1.30-4.02; *P*=.004) and age group (20-24 years) were statistically significantly associated with women’s ability to recall the short code correctly (OR 1.81, 95% CI 1.08-3.04; *P*=.02). Women from districts A (OR 3.88, 95% CI 1.50-10.00; *P*=.005), B (OR 2.39, 95% CI 1.17-4.86; *P*=.01), and D (OR 3.19, 95% CI 1.25-8.15; *P*=.01) had higher odds of expressing higher satisfaction than women from district E.

**Table 3:** Predictors of women’s adoption of mobile health service.

Variables			High satisfaction				Recalled short code		
			Adjusted OR^a^ (95% CI)		*P* value		Adjusted OR (95% CI)		*P* value
**Who accessed messages**									
	Women	1.72 (1.12-2.63)		.01		2.88 (1.90-4.36)		<.001	
	Women or family member (reference)	1 (N/A^b^)		N/A		1 (N/A)		N/A	
**Age of respondents (years)**									
	<20	0.88 (0.43-1.85)		.89		1.01 (0.50-2.03)		.97	
	20-24	0.84 (0.49-1.43)		.84		1.81 (1.08-3.04)		.02	
	≥25 (reference)	1 (N/A)		N/A		1 (N/A)		N/A	
**Educational qualification of women**									
	No/primary (reference)	1 (N/A)		N/A		1 (N/A)		N/A	
	Junior secondary	0.96 (0.58-1.59)		.88		1.17 (0.72-1.90)		.51	
	Secondary or higher	1.08 (0.60-1.92)		.80		2.28 (1.30-4.02)		.004	
**Family monthly income, BDT^c^** **(US $)**									
	≤10,000 (118.15; reference)	1 (N/A)		N/A		1 (N/A)		N/A	
	10,001-20,000 (118.15-236.30)	1.96 (1.24-3.11)		.004		1.44 (0.92-2.26)		.11	
	>20,000 (236.30)	2.48 (1.25-4.93)		.009		1.66 (0.86-3.20)		.13	
**First-time pregnancy**									
	Yes	1.12 (0.66-1.89)		.66		1.10 (0.65-1.80)		.71	
	No (reference)	1 (N/A)		N/A		1 (N/A)		N/A	
**Number of** **antenatal care** **visits**									
	>4	2.86 (1.82-4.48)		<.001		1.17 (0.75-1.82)		.48	
	≤4 (reference)	1 (N/A)		N/A		1 (N/A)		N/A	
**Place of residence**									
	Urban	0.56 (0.29-1.09)		.09		1.34 (0.74-2.45)		.33	
	Rural (reference)	1 (N/A)		N/A		1 (N/A)		N/A	
**Districts**									
	District A	3.88 (1.50-10.00)		.005		1.49 (0.65-3.44)		.35	
	District B	2.39 (1.17-4.86)		.01		0.99 (0.52-1.93)		.99	
	District C	1.70 (0.73-3.95)		.16		1.08 (0.48-2.41)		.85	
	District D	3.19 (1.25-8.15)		.01		1.44 (0.60-3.44)		.41	
	District E (reference)	1 (N/A)		N/A		1 (N/A)		N/A	

^a^OR: odds ratio.

^b^Not applicable.

^c^BDT: Bangladeshi Taka.

### Effect on Readiness for Pregnancy and Delivery Complications

The type of access to messages did not show a statistically significant effect on women’s readiness for pregnancy and delivery complications (Table 4). However, completion of secondary education was associated with women’s ability to recall danger signs (OR 2.44, 95% CI 1.40-4.26; *P*=.001) and arrange skilled delivery (OR 2.37, 95% CI 1.05-5.32; *P*=.02) and blood donors (OR 5.33, 95% CI 1.74-16.32; *P*=.003). Similarly, women who received more than 4 ANC visits had higher odds of showing the expected behavioral outcomes (danger signs: OR 1.86, 95% CI 1.21-2.86; *P*=.004; skilled delivery: OR 2.13, 95% CI 1.15-3.96; *P*=.02; and blood donor: OR 3.71, 95% CI 1.52-9.02; *P*=.004) than women who had lower number of ANC visits. In addition, women from higher-income households had higher odds of recalling danger signs (OR 3.15, 95% CI 1.63-6.06; *P*=.001) and selecting blood donors (OR 4.48, 95% CI 1.42-14.08; *P*=.01) than women from the lower-income households. Age was statistically significantly associated with women’s delivery decisions; pregnant women who were aged less than 20 years (OR 0.17, 95% CI 0.06-0.47; *P*=.001) or aged 20 to 24 years (OR 0.31, 95% CI 0.15-0.63; *P*=.001) had lower odds of planning delivery with skilled birth attendant at home or a hospital than women who were aged 25 years or older. District was a confounder for 2 behavioral outcomes; women residing in district B had higher odds of planning skilled delivery (OR 4.59, 95% CI 1.77-11.90; *P*=.002) and arranging blood donors (OR 10.87, 95% CI 2.97-39.78; *P*<.001) than women from district E.

**Table 4:** Predictors of subscribers’ readiness for pregnancy and delivery complications.

Variables		Able to recall danger signs		Planned skilled delivery			Selected blood donor for delivery and emergency	
		Adjusted (OR^a^ 95% CI)	*P* value	Adjusted (OR 95% CI)	*P* value	Adjusted (OR 95% CI)		*P* value
**Who accessed messages**								
	Women	1.07 (0.71-1.61)	.71	0.76 (0.42-1.37)	.37	1.62 (0.70-3.75)		.25
	Women or family member (reference)	1 (N/A^b^)	N/A	1 (N/A)	N/A	1 (N/A)		N/A
**Age of respondents (years)**								
	<20	0.74 (0.37-1.47)	.40	0.17 (0.06-0.47)	.001	1.34 (0.35-5.06)		.66
	20-24	0.81 (0.48-1.35)	.43	0.31 (0.15-0.63)	.001	0.87 (0.34-2.21)		.77
	≥25 (reference)	1 (N/A)	N/A	1 (N/A)	N/A	1 (N/A)		N/A
**Educational qualification of women**								
	No/primary (reference)	1 (N/A)	N/A	1 (N/A)	N/A	1 (N/A)		N/A
	Junior secondary	0.99 (0.62-1.59)	.97	1.34 (0.66-2.71)	.34	1.54 (0.54-4.44)		.41
	Secondary or higher	2.44 (1.40-4.26)	.001	2.37 (1.05-5.32)	.02	5.33 (1.74-16.32)		.003
**Family monthly income, BDT^c^** **(US $)**								
	≤10,000 (118.15; reference)	1 (N/A)	N/A	1 (N/A)	N/A	1 (N/A)		N/A
	10,001-20,000 (118.15-236.30)	2.08 (1.35-3.21)	.001	0.89 (0.47-1.70)	.74	1.86 (0.72-4.77)		.19
	>20,000 (236.30)	3.15 (1.63-6.06)	.001	1.52 (0.62-3.68)	.35	4.48 (1.42-14.08)		.01
**First-time pregnancy**								
	Yes	0.86 (0.52-1.41)	.55	1.61 (0.80-3.20)	.17	1.18 (0.46-3.03)		.72
	No (reference)	1 (N/A)	N/A	1 (N/A)	N/A	1 (N/A)		N/A
**Number of** **antenatal care** **visits**								
	>4	1.86 (1.21-2.86)	.004	2.13 (1.15-3.96)	.016	3.71 (1.52-9.02)		.004
	≤4 (reference)	1 (N/A)	N/A	1 (N/A)	N/A	1 (N/A)		N/A
**Place of residence**								
	Urban	1.00 (0.55-1.80)	.99	0.70 (0.32-1.55)	.38	0.45 (0.16-1.20)		.11
	Rural (reference)	1 (N/A)	N/A	1 (N/A)	N/A	1 (N/A)		N/A
**Districts**								
	District A	0.80 (0.36-1.81)	.60	0.82 (0.25-2.66)	.74	2.21 (0.43-11.27)		.33
	District B	1.11 (0.58-2.13)	.74	4.59 (1.77-11.90)	.002	10.87 (2.97-39.78)		<.001
	District C	1.14 (0.51-2.52)	.74	0.94 (0.30-2.87)	.91	0.45 (0.08-2.46)		.36
	District D	1.39 (0.59-3.29)	.44	1.56 (0.43-5.64)	.49	2.02 (0.38-10.62)		.40
	District E (reference)	1 (N/A)	N/A	1 (N/A)	N/A	1 (N/A)		N/A

^a^OR: odds ratio.

^b^Not applicable.

^c^BDT: Bangladeshi Taka.

### Effect on Maternal Wellness Behavior at the Household Level

Women’s sole access to mobile phone–based educational messages showed a statistically significant effect on the availability of nutritional elements 5 times a day (OR 1.58, 95% CI 1.04-2.40; *P*=.03) and access to potable drinking water (OR 1.90, 95% CI 1.17-3.09; *P*=.01; Table 5). Whether women solely accessed phone messages did not show a statistically significant association with their hand washing practices.

Higher family income (OR 2.40, 95% CI 1.25-4.61; *P*=.008) and more than 4 ANC visits (OR 3.46, 95% CI 2.22-5.40; *P*<.001) were other determinants of women’s improved food consumption behavior (Table 5). District was a confounder for pregnant women’s self-reported wellness behavioral outcomes at home; women from districts C (hand washing: OR 3.24, 95% CI 1.15-9.12; *P*=.02) and D (nutrition: OR 0.31, 95% CI 0.13-0.74; *P*=.008; drinking water: OR 7.26, 95% CI 2.78-18.9; *P*<.001; and hand washing: OR 10.73, 95% CI 3.75-30.63; *P*<.001) had higher odds of showing improved behavior than women from district E.

**Table 5:** Predictors of following maternal wellness instructions at the household level.

Variables		Consumed nutritious food 5 or more times per day		Followed Aponjon messages on drinking water			Followed Aponjon messages on hand washing		
		Adjusted OR^a^ (95% CI)	*P* value		Adjusted OR (95% CI)	*P* value		Adjusted OR (95% CI)	*P* value
**Who accessed messages**									
	Women	1.58 (1.04-2.40)	.03		1.90 (1.17-3.09)	.01		1.31 (0.80-2.13)	.28
	Women or family member (reference)	1 (N/A^b^)	N/A		1 (N/A)	N/A		1 (N/A)	N/A
**Age of respondents (years)**									
	<20	1.72 (0.85-3.48)	.13		1.19 (0.54-2.60)	.66		1.03 (0.46-2.34)	.93
	20-24	1.11 (0.66-1.86)	.68		0.91 (0.51-1.64)	.77		0.82 (0.44-1.50)	.52
	≥25 (reference)	1 (N/A)	N/A		1 (N/A)	N/A		1 (N/A)	N/A
**Educational qualification of women**									
	No or primary (reference)	1 (N/A)	N/A		1 (N/A)	N/A		1 (N/A)	N/A
	Junior secondary	1.52 (0.93-2.49)	.09		0.87 (0.49-1.53)	.63		0.82 (0.46-1.48)	.52
	Secondary or higher	1.73 (0.99-3.03)	.05		0.80 (0.42-1.55)	.51		0.66 (0.34-1.28)	.22
**Family monthly income, BDT^c^** **(US $)**									
	≤10,000 (118.15; reference)	1 (N/A)	N/A		1 (N/A)	N/A		1 (N/A)	N/A
	10,001-20,000 (118.15-236.30)	1.53 (0.98-2.40)	.06		1.25 (0.74-2.09)	.39		1.43 (0.85-2.42)	.17
	>20,000 (236.30)	2.40 (1.25-4.61)	.008		0.80 (0.38-1.66)	.55		0.57 (0.24-1.31)	.18
**First-time pregnancy**									
	Yes	0.87 (0.52-1.44)	.60		1.20 (0.68-2.12)	.51		0.95 (0.53-1.71)	.87
	No (reference)	1 (N/A)	N/A		1 (N/A)	N/A		1 (N/A)	N/A
**Number of** **antenatal care** **visits**									
	>4	3.46 (2.22-5.40)	<.001		0.94 (0.58-1.52)	.80		0.66 (0.40-1.09)	.11
	≤4 (reference)	1 (N/A)	N/A		1 (N/A)	N/A		1 (N/A)	N/A
**Place of residence**									
	Urban	1.26 (0.69-2.31)	.44		1.42 (0.73-2.74)	.29		1.59 (0.78-3.21)	.19
	Rural (reference)	1 (N/A)	N/A		1 (N/A)	N/A		1 (N/A)	N/A
**Districts**									
	District A	0.89 (0.39-2.06)	.79		0.44 (0.13-1.47)	.18		1.49 (0.49-4.52)	.47
	District B	0.52 (0.26-1.02)	.06		2.23 (1.01-4.94)	.04		1.74 (0.69-4.38)	.23
	District C	0.62 (0.28-1.40)	.26		1.92 (0.75-4.92)	.17		3.24 (1.15-9.12)	.02
	District D	0.31 (0.13-0.74)	.008		7.26 (2.78-18.9)	<.001		10.73 (3.75-30.63)	<.001
	District E (reference)	1 (N/A)	N/A		1 (N/A)	N/A		1 (N/A)	N/A

^a^OR: odds ratio.

^b^Not applicable.

^c^BDT: Bangladeshi Taka.

## Discussion

### Principal Findings

We evaluated the association between women’s unequal access to mobile phones and their perception about using mobile phone–based health services for pregnancy and birth preparedness in their socioeconomic context. Understanding the socioeconomic factors that contribute to women’s access to mHealth services has implementation benefits in low-income settings where sharing a single phone among family members is a common phenomenon [4,14,15].

Our findings suggest that women’s differential access to targeted messages positively affects their satisfaction and familiarity with the service short code and the adoption of certain recommended practices around pregnancy wellness, such as inclusion of nutritious elements in regular diets and drinking potable water. These findings were consistent when the models were adjusted for all covariates (age, education, income, first pregnancy, ANC frequency, and place of residence) and district. These findings confirm 2 aspects of the adoption of mobile phone–based educational service for pregnancy: (1) *sole and uninterrupted access to messages* provide target women an understanding of the service before they decide to adopt or reject a new innovation (mHealth) [39,40] and (2) women who access messages by themselves are in a better position to evaluate the context, need, complexity, and relative advantage of the information provided by mobile phones than women who occasionally or never access messages [39]. Our findings also suggest that women’s age, education, and family income were statistically significantly associated with their mHealth experience. Although national initiatives and investment to improve employment opportunities and secondary school education should continue, mHealth implementers could ensure equity in accessing messages among subscribers by facilitating equitable approaches such as lending mobile phones to women from underprivileged households for the entire service period and program-specific training of clients, especially older women (aged ≥25 years) and women who have not completed secondary education, on operating mobile phones and accessing messages at the established times. We recommend additional equitable approaches, such as mHealth-supported cash vouchers for pregnant women and infants [41], to address prevailing food shortages in underprivileged households [42]. Furthermore, mobile phone–based messaging services have the potential to increase awareness of public health problems such as water-borne diseases and arsenic contamination in groundwater [38].

In our study, women’s differential access to messages did not affect their choice of skilled birth attendant at home or a hospital, knowledge about the danger signs of pregnancy, and arrangement of blood donors for delivery and complications. Instead, we found, similar to previous studies, higher frequency of ANC visits and women’s privileged background to have significantly improved maternal knowledge about the danger signs and adoption of BPCR measures [9,43]. We need to evaluate these findings carefully, as these behavioral outcomes may have been influenced by existing national campaigns and overall improvements in the utilization of maternal and child health care facilities [19,28,44]. For example, the rate of facility-based delivery has almost doubled from 23% in 2010 to 47% in 2016 [45], which could be a result of the government-funded voucher schemes to support marginalized pregnant women to have facility-based deliveries, checkups, tests, and arrangement of transport to facilities at free of cost [44]. Our recent exploratory study suggested that women, in general, were well informed about delivery and postpartum care guidelines by their community health workers, hospital staff, and national media during their regular ANC visits and were rather interested in receiving information on newborn care and nutrition from Aponjon service [46]. The study also suggested that although women from higher-income households could afford services at private hospitals, home-based deliveries were usually preferred by low-income households for uncomplicated births because of convenience, cost, and fear of C-section and were organized by elderly female family members and local (untrained) birth attendants [46,47].

Our findings are concerning though as younger women, especially who were in their late teens, did not have a plan for delivery with skilled birth attendant or at a facility. The national survey estimates that pregnancy-related mortality ratio among 15- to 19-year-old women has almost doubled from 75 deaths in 2010 to 144 deaths per 100,000 live births in 2016 and that first-time mothers are at increased risk of maternal deaths (215 per 100,000 live births) compared with mothers with live children [45]. Educating a large population of teenaged pregnant women on safe motherhood is challenging for the Bangladeshi government, especially in a situation where one-third of 15- to 19-year-old girls are likely to experience pregnancy before reaching adulthood [9,48]. Hence, the importance of repeat visits and family counseling of community health workers during pregnancy in low-income households, especially for adolescent women who are likely to rely on husbands and mothers-in-laws for delivery decisions, is undeniable [25,43,48,49].

Our research is limited by a number of factors. First, the results would be strengthened by a randomized control trial or pre-post quasi-experimental design rather than a cross-sectional survey. Small differences in the behavioral outcome associated with women’s type of access could not be captured because of the power of the study that was designed a priori based on a moderate to large association; the study required a larger sample size for greater generalizability of our findings. Second, the study relied on respondents’ self-reported behavioral outcomes rather than their attendance report at facilities or actual habits, and there may be problems with recall bias and overreporting. Third, the study could have benefited from system-generated data on the actual number of messages accessed by these 2 different groups of respondents, as a study in the United States reported a positive association between access to high frequency of messages and abstinence from alcohol consumption during the postpartum period [50].

### Way Forward

Nevertheless, our research will be helpful for mHealth implementers who are working to reduce maternal and neonatal deaths in developing countries. Our research confirms that *women’s sole access to messages* can change their perception of the service and adoption of maternal wellness messages. We infer from our findings that irrespective of how women access messages, one-way (voice) messages alone may not improve delivery decisions, which are controlled by socioeconomic circumstances of individual families and their personal experiences [46]. Therefore, mHealth implementers working in resource-limited settings need to consider cultural and socioeconomic constraints that limit women’s access to mHealth services and all other maternal health care services and should adopt a holistic approach to ensure equity in their service. ANC visits (more than 4) remain an independent factor for women’s knowledge about pregnancy danger signs, birth preparedness, and maternal dietary diversity. mHealth services such as Aponjon may consider introducing mobile phone apps for health workers and a linked referral system to increase their efficiency in identifying high-risk mothers during monthly door-to-door ANC visits, maintaining follow-up visits, minimizing workload, and referring pregnant women to proper facilities [18,51-54]. Future studies should include anthropometric measurement and hospital data to understand the effect of pregnancy messages on birth outcomes [55,56].

### Conclusions

Socioeconomic and cultural barriers to mobile phone access by women can be problematic for implementing mHealth services in resource-limited settings. Although the national policy targeting poverty alleviation and women’s empowerment requires revisions, stand-alone mHealth implementers need to integrate with local health infrastructure and invest in building women’s capacity to access mobile phones. We suggest ongoing research to monitor temporal trends in women’s differential access to mobile phone messages in South Asian countries.

## Abbreviations

ANC: antenatal care
ANU: Australian National University
BDT: Bangladeshi Taka
BPCR: birth preparedness and complication readiness
IRB: international institutional review board
IVR: interactive voice resonance
mHealth: mobile health
MMR: maternal mortality ratio
NMR: neonatal mortality rate
OR: odds ratio
UP: union parishad
VIF: variance inflation factor
WHO: World Health Organization
